# Precise and low-power closed-loop neuromodulation through algorithm-integrated circuit co-design

**DOI:** 10.3389/fnins.2024.1340164

**Published:** 2024-03-14

**Authors:** Jie Yang, Shiqi Zhao, Junzhe Wang, Siyu Lin, Qiming Hou, Mohamad Sawan

**Affiliations:** CenBRAIN Neurotech, School of Engineering, Westlake University, Hangzhou, China

**Keywords:** closed-loop, implantable devices, neuromodulation, artificial intelligence, ASIC, event-driven, low-power

## Abstract

Implantable neuromodulation devices have significantly advanced treatments for neurological disorders such as Parkinson’s disease, epilepsy, and depression. Traditional open-loop devices like deep brain stimulation (DBS) and spinal cord stimulators (SCS) often lead to overstimulation and lack adaptive precision, raising safety and side-effect concerns. Next-generation closed-loop systems offer real-time monitoring and on-device diagnostics for responsive stimulation, presenting a significant advancement for treating a range of brain diseases. However, the high false alarm rates of current closed-loop technologies limit their efficacy and increase energy consumption due to unnecessary stimulations. In this study, we introduce an artificial intelligence-integrated circuit co-design that targets these issues and using an online demonstration system for closed-loop seizure prediction to showcase its effectiveness. Firstly, two neural network models are obtained with neural-network search and quantization strategies. A binary neural network is optimized for minimal computation with high sensitivity and a convolutional neural network with a false alarm rate as low as 0.1/h for false alarm rejection. Then, a dedicated low-power processor is fabricated in 55 nm technology to implement the two models. With reconfigurable design and event-driven processing feature the resulting application-specific integrated circuit (ASIC) occupies only 5mm^2^ silicon area and the average power consumption is 142 μW. The proposed solution achieves a significant reduction in both false alarm rates and power consumption when benchmarked against state-of-the-art counterparts.

## 1 Introduction

**Figure 1:**
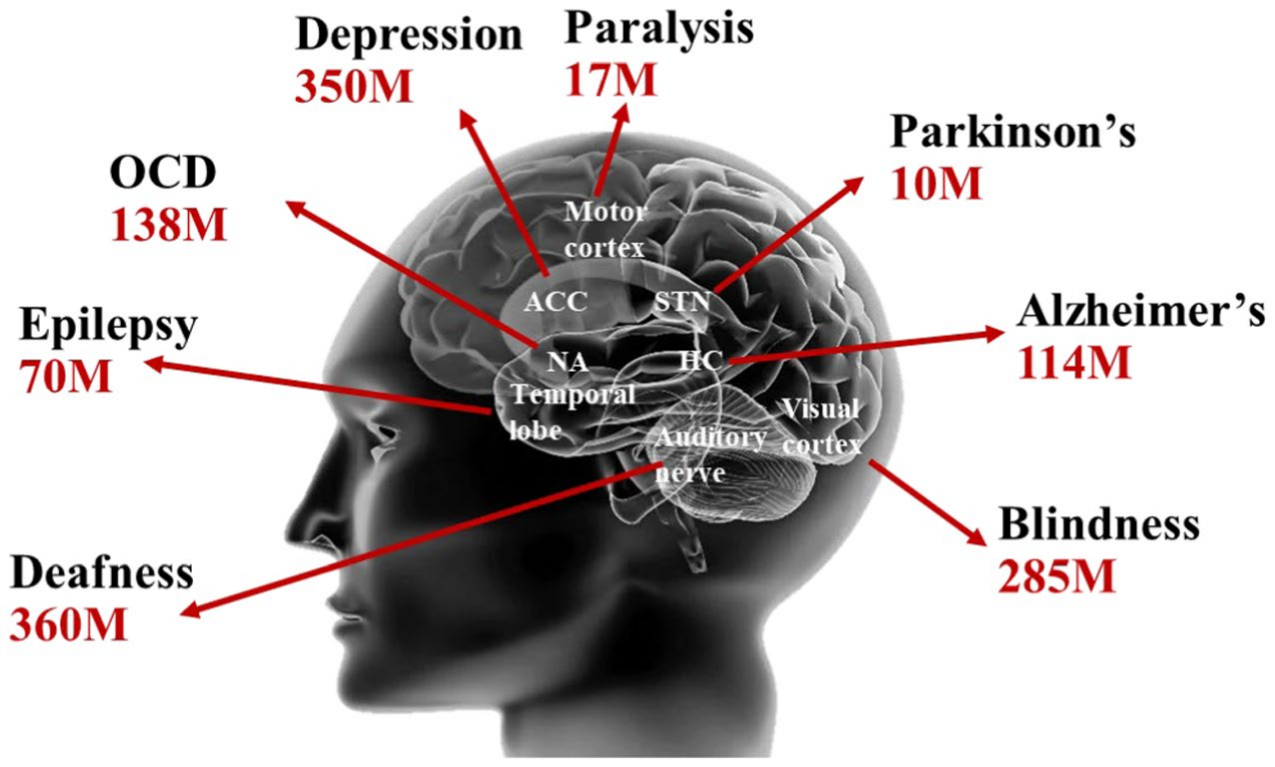
Brain disease statistics from WHO (Lee et al., 2020).

With the prolongation of human life expectancy and the emerging of the aging society, brain disorders such as epilepsy, Parkinson’s disease, depression have inflicted suffering on a significant portion of the global population. Brain disorders not only pose a severe threat to human health but also impose a substantial medical and societal burden, ranking as the leading cause of all diseases (Poo et al., 2016). As shown in Figure 1, the latest statistics from the World Health Organization (WHO) indicate that the numbers of individuals affected by brain disorders such as epilepsy, Parkinson’s disease, and depression have exceeded 70, 10, and 350 million, respectively (Lee et al., 2020).

Traditional treatments for brain disorders primarily involve medication and surgical procedures. However, medication-based treatments often come with significant side effects, slow progress, and the risk of developing drug resistance. For instance, approximately 30% of epilepsy patients exhibit drug resistance or adverse reactions. Moreover, irreversible surgeries can lead to unpredictable adverse consequences for patients, including impairments in memory, vision, and motor function (Kuhlmann et al., 2018b).

**Figure 2:**
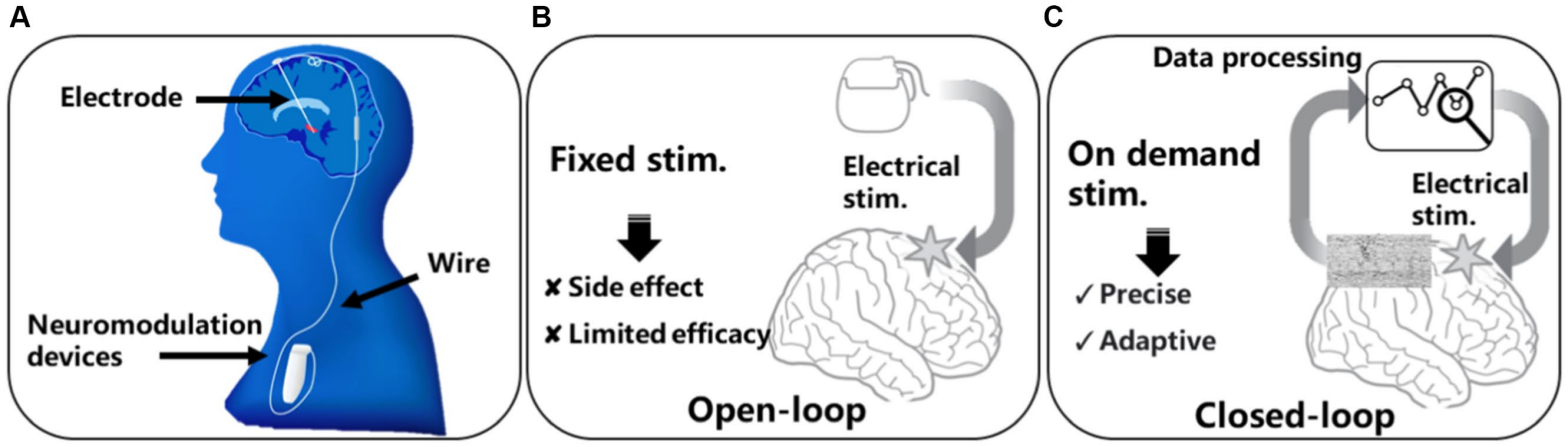
Neuromodulation modalities: **(A)** conventional DBS device, **(B)** open-loop neuromodulation, **(C)** closed-loop neuromodulation.

In recent years, the utilization of implantable medical devices for neuromodulation has emerged as one of the most effective approaches for treating various brain disorders, and it has benefited hundreds of thousands of brain disorder patients worldwide (Won et al., 2020). Figure 2A illustrates the forms and implantation of current neuromodulation devices. The devices are typically implanted in the chest region through invasive surgeries and connected to electrodes implanted near the target brain regions via wires. Most neuromodulation systems employ an open-loop design, as depicted in Figure 2B, where the device delivers continuous or periodically stimulation to the nerves. For example, deep brain stimulation (DBS) for Parkinson’s treatment can deliver electrical stimulation to target brain regions to regulate the faulty nerve signals causing tremors, rigidity, and other symptoms. Although these open loop systems provide possibilities of the treatment of many brain disorders, the lack of adaptability to the dynamic neural activity or the changing needs of the patients have limited their ability to deliver personalized and optimal treatment outcomes. Moreover, open-loop control suffers from inefficiency because continuous nerve stimulation can lead to habituation and neurological chemical changes, resulting in a decrease in treatment efficacy and safety issues. Side-effect such as dyskinesia have been constantly reported in epilepsy and Parkinson’s patients due to open-loop stimulations (Dembek et al., 2017).

To address the issues associated with open-loop control, a natural solution is to monitor the activity of the nervous system in real-time and determine whether disease-related features exist in the neural signals before initiating nerve stimulation. This approach is known as closed-loop control, as illustrated in Figure 2C. Closed-loop systems integrate real-time data processing and responsive stimulation functions, enabling a more sophisticated and adaptive approach to neuromodulation. By continuously monitoring neural activity and adapting stimulation parameters accordingly, closed-loop neuromodulation devices offer enhanced precision, efficacy, and patient-specific therapy (Scangos et al., 2021). Algorithms used for biomarker detection are crucial for achieving closed-loop neuromodulation. Currently, the algorithms primarily used in closed-loop neuromodulation systems main involves neural signal features extraction and classification. Features such as Phase Locking Value (PLV; O'Leary et al., 2018), Spectrum Energy (SE; Cheng et al., 2018; O'Leary et al., 2018; Huang et al., 2019; Zhang et al., 2022), line length (Shin et al., 2022) that can reflect the amplitude, phase, and frequency of the neural signal are commonly used. The classification mainly relies on linear regression and support vector machine classifiers. However, due to limited on-device battery and computing resource, the algorithms used in existing neuromodulation devices still suffer from a high false positive rate. The reported false positive can exceeds 2,800 per day (Bruno et al., 2020), which greatly undermining the effectiveness of the closed-loop neuromodulation.

In recent years, artificial neural networks have begun to reshape closed-loop neuromodulation, substantially reducing false positives. In 2018, an epilepsy detection algorithm that used short-time Fourier features and convolutional neural networks has been reported (Truong et al., 2018). The algorithm has reduced the false positive rate to below 0.21 fp/h, but due to the use of large convolutional kernels and neural network with over seven layers, the model’s parameter size reached 0.76 MB, the associated computation exceeds the capacity of implant medical devices. In 2019, a comparative study of different classification algorithms was conducted, validating the superiority of neural networks in closed-loop control (Daoud and Bayoumi, 2019). In 2020, the difference between regression, SVM and CNN for closed-loop control was reviewed and compared in Yang and Sawan (2020). An approach that combined direct transfer functions and neural networks reduced the false positive rate to 0.08/h, but it involved a large number of multiplicative and convolutional calculations, and the model size exceeded 1 MB (Wang et al., 2020). Recently, research like EEGNet (Lawhern et al., 2018) and its variant (Schneider et al., 2020) have been proposed to optimize the neural network so that can be used in embedded systems. Through the network size is reduce, they still cannot be migrated to implantable devices with limited storage and computing capacity.

To solve the computing and power consumption issues associate with the close-loop control, dedicated integrated signal processing chips have been developed. A closed-loop chip with a power consumption of 3.12 mW and an area of 25mm^2^ was reported. Frequency features and linear regression classification method achieved a sensitivity of 97.8% and a false positive rate of 2 fp/h (Cheng et al., 2018). O'Leary et al. (2018) reported an integrated chip with a sensitivity of 100%, and a false positive rate of 0.81 fp/h. The chip had an area of 7.6 mm^2^ and consumed 1.07 mW. In 2019, a dedicated neural signal processing chip with 4.5 mm^2^ area and 1.9 mW power consumption was reported (Huang et al., 2019). It achieved a sensitivity of 96.6% and a false positive rate of 0.28 fp/h. Recently, an integrated chip with an area of 4.5 mm^2^ and power consumption of 1.2 mW, achieving a sensitivity of 97.8% and a false positive rate of 0.5 fp/h was reported (Zhang et al., 2022). Recently, a SVM-based processor has been proposed and achieved 92.0% sensitivity and 0.57/h false alarm rate in seizure prediction task (Hsieh et al., 2022). With optimized SVM algorithm and customized circuits implementation, the chip consumes less than 4 mm^2^ silicon area, and the power is reduced to 2.3 mW, exhibit reduced power consumption when compared to embedded microprocessors for the computing of the same complexity.

In this study, we undertake an algorithm-integrated circuit co-design approach for close-loop control with dedicated integrated circuits to address the issues related to false alarm rates and power consumption. Initially, we employ a neural-network search strategy to acquire neural network models that not only demand less computational effort but also exhibits low false alarm rate. These models are then quantized to minimal memory and computation resource requirements. A low-power, event-driven processor was designed to implement these models, allowing consistently monitor events in a low-power state and transition to a high-precision state for eliminating false alarms. The performance and the efficiency of the proposed method are validated with a real-time seizure prediction demonstration system.

The organization of this paper is as following. Section 2 describes the detail of the algorithm design and the optimization strategies. Chip architecture and circuit design are given in section 3. Experiments and evaluation results are summarized in Section 4. The last section concludes this paper.

## 2 Algorithm designs

To minimize the false positive rate and power consumption, our study adopts a two-stage optimization approach. Initially, a network search space is defined, with consideration of the sensitivity and false positive rate requirements specific to closed-loop neuromodulation. A targeted network search strategy yields a baseline model which meets these criteria. Subsequently, the baseline model is refined using network quantization techniques, which reduces the model to sizes and computational requirement appropriate for integration within the limited resources of implantable systems.

### 2.1 Network search

**Figure 3:**
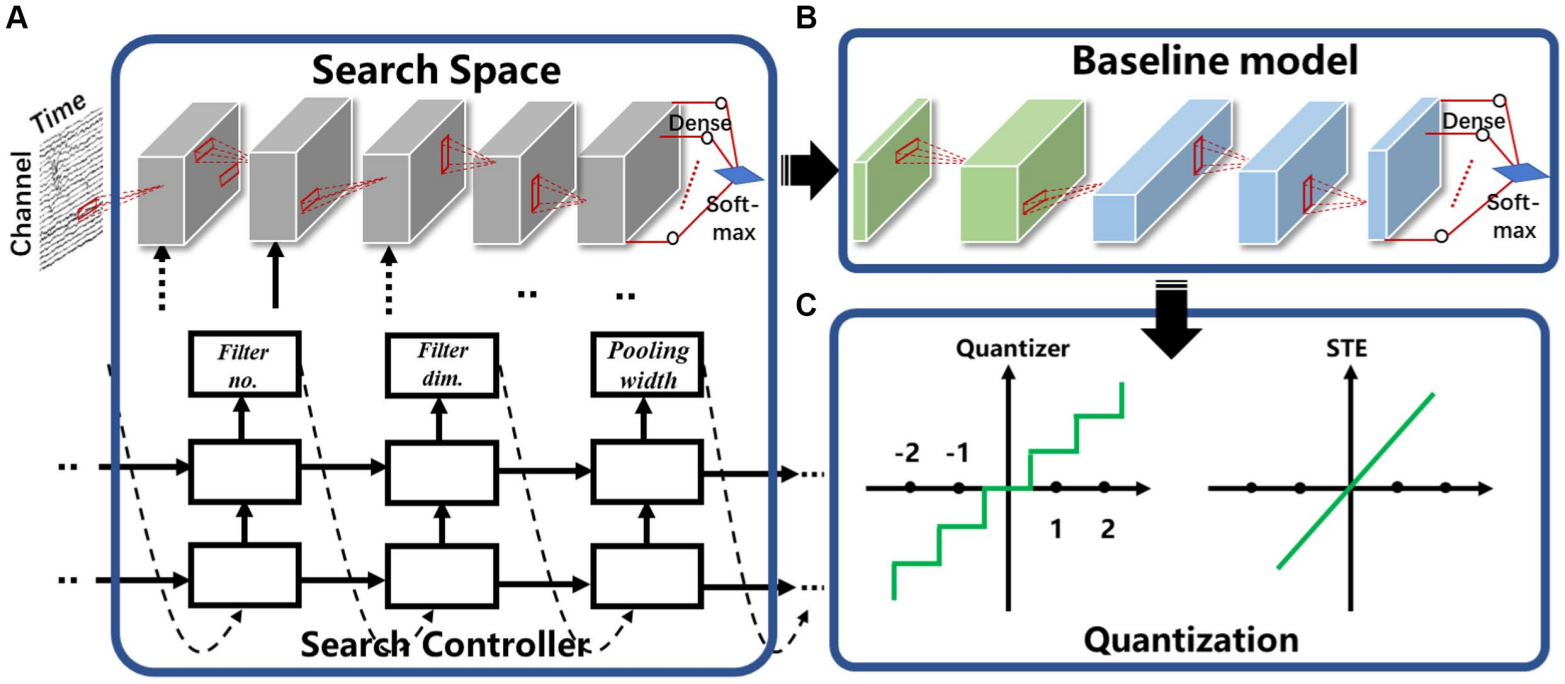
The proposed model compression strategy: **(A)** search space to generate baseline model with high sensitivity and low false alarm rate, **(B)** baseline model with the first two block for temporal information extraction and the last three block for spatial convolution, **(C)** quantization and straight through estimators (STE).

Traditional neural signal processing techniques like line length, although simple to implement in hardware, exhibit limited accuracy in recognition. Consequently, they result in a high false positive rate during closed-loop control processes, making them unsuitable for precise treatment. On the other hand, currently available highly accurate and low false alarm rate algorithms rely on neural networks, but their sizes typically exceed several hundreds of kilobytes (kB), which surpasses the storage capacity and computing resource of many implantable chips. In this study, we first define an approximate network structure space (Figure 3A) based on the available on-chip storage and computational resources of implantable chips (Yang and Sawan, 2020; Martínez et al., 2022). In neuromodulation systems, there are a few to tens of channels, and each channel is sampling at the rate of at least few hundred Hertz. Hence, the sampled data has extremely unbalanced X (time) axis and Y axis (channel), and the Y axis would dimmish rapidly if 2d convolution is applied in the beginning. Moreover, it may decrease the classification performance if the time (X) and channel (Y) axis are mixed up (Zhao et al., 2020; Wang et al., 2021). Therefore, a channel-wise neural network structure is proposed in this work to avoid mixing up data features from different channels. Using one-dimensional convolutions, data processing occurs independently within each channel, ensuring that there is no mixing or interaction of data across channels during these operations. It also reduces memory and computing overhead for hardware implementation.

As depicted in Figure 3B, the network structure comprises five distinct blocks, the first three blocks are responsible for temporal information extraction, while the latter two blocks perform spatial convolution. Each convolutional block consists of a convolutional layer and a pooling layer, with a Batch Normalization (BN) layer and a rectified linear unit (ReLU) activation function applied after the convolutional layer. The global average pooling layer is applied to further reduce the number of parameters. The finial output was obtained using a dense layer with the Softmax function. During network search, the convolution kernels are restricted to one-dimensional to reduce the number of parameters and associated computation. For the first three blocks that operate on the spatial dimension, filter and pooling height are fixed as 1, and an RNN-based search controller is employed to explore the sizes, quantities, and pooling widths of the convolution kernels. It selects filter width from the options of [1, 2, 8, 16], the number of filters from the choices of [4, 8, 16], and select a pooling width from the set [1, 4, 8, 16]. For the last two convolutional blocks, which operated on the channel dimension, the filter width and pooling width were fixed at 1. Similarly, the controller RNN also make decisions regarding filter height, which could be chosen from [1, 2, 8, 16], the number of filters (from [4, 8, 16]), and the pooling height (from [1, 2, 4]). All the strides of the convolutional layer were fixed as 1. The RNN controller generates a description of the target neural network, including the number and dimensions of convolution kernels and pooling widths. Once the RNN controller completes the generation of the description string, the neural network it represents is constructed, trained, and validated. The validation results are used as a reward signal to update the RNN controller, and reinforcement learning is used to maximize the expected reward function J(ω), defined as Eq. 1:

(1) $$J\left(\omega \right)=E_{P\left(a_{1:T};\omega \right)}\left[R\right]$$

*α*_1:T_ represents the string generated by the RNN controller, following the probability distribution *P*. *R* is the validation accuracy of the network generated by the controller, serving as the reward signal for training the controller. We update the RNN controller parameters using the following reinforcement learning approach of Eq. 2 (Williams, 1992):

(2) $$\frac{1}{K}\overset{K}{\underset{k=1}{\sum }}\overset{T}{\underset{t=1}{\sum }}\nabla \mathrm{\omega logP}\left(a_{t}|a_{\left(t-1\right):1};\omega \right)\left(R_{k}-b\right)$$

*K* is the number of network structures generated by the RNN controller in a batch, and *T* is the number of hyperparameters predicted by the controller in the string. *R_k_* represents the accuracy of the *k*-th network structure, and *b* is the exponentially moving average of the accuracy of the previous architecture. By constraining the search space, we can identify the best-performing model under the specified conditions as the baseline model for further compression.

### 2.2 Baseline model compression

Network quantization represents a highly effective approach for compressing and accelerating neural networks. In this method, the neural network’s weights and activation values, originally stored as high-bit floating-point numbers, are converted into low-bit integers or fixed-point numbers. This transformation results in a reduction in the number of operations required by the neural network and lowers the hardware implementation costs, as evidenced by previous studies (Alyamkin et al., 2019). In some extreme cases, parameters of a neural network can be quantized to just 1-bit, taking values of −1 or 1 (Courbariaux et al., 2016). This leads to a significant reduction in multiply-accumulate operations, which are replaced by the more efficient 1-bit XNOR operation in the hardware. While this reduces the amount of memory required for access, it is worth noting that this extreme quantization can lead to certain performance compromises, including increasing false alarm rate and diminished sensitivity.

In our study, we assess the performance of different bit quantization techniques in the context of epileptic seizure prediction networks. We aim to determine if the reduction in performance, resulting from these quantization methods, falls within acceptable limits. We utilize fixed-point representation to quantize both weights and activations to the same number of bits. Specifically, we employ the weight quantization method described in Eq. 3 (Moons et al., 2017).

(3) $$q=\mathrm{clip}\left(\frac{\mathrm{round}\left(2^{Q-1}\times \omega \right)}{2^{Q-1}},-1,\;1-2^{-\left(Q-1\right)}\right)$$

Here, ω represents the weights before quantization, and Q denotes the number of bits required for the quantization process. In the quantization of activations, we utilize the above quantized *tanh* function. Figure 3C shows the quantization process. When training the quantized model, the gradient propagated by the straight-through estimators (STE) uses function Eq. 4 (Courbariaux et al., 2016) regardless of how the number of quantized bits Q was

(4) Htanha=Clipa,−1,1=max−1,min1,a

This function is used because the quantization is a nondifferentiable operation during the backward pass.

## 3 Chip design

**Figure 4:**
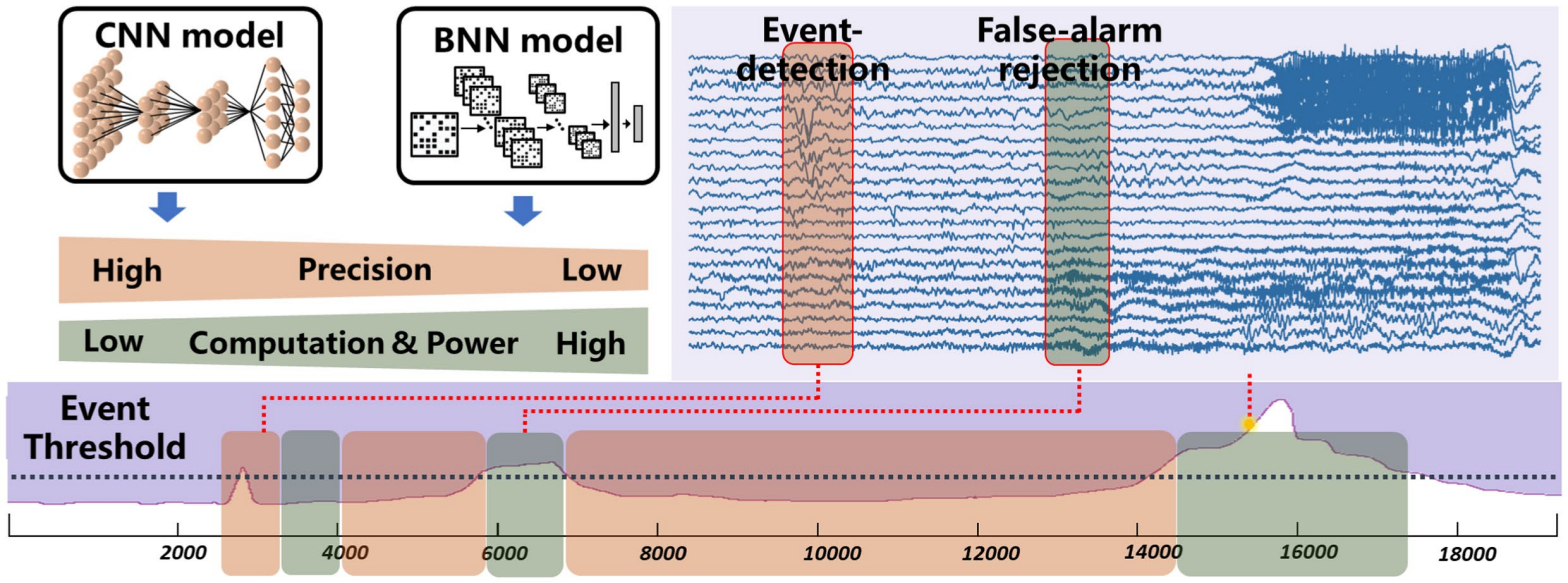
Motivation of the proposed event-driven neural signal processor.

Complex neural signal processing is the primary contributor to system power consumption. While common low-power technologies like periodic wake-up and frequency reduction can decrease power usage, they come at the cost of compromising real-time performance in closed-loop neuromodulation. The proposed chip features low-power, event-driven real-time processing by exploiting the sparsity inherent in brain disease occurrences. Figure 4 demonstrates the motivation behind the event-driven processing approach. Neural signals typically exhibit a consistent pattern and lack distinct characteristics most of the time. Devices designed for detecting or predicting disease biomarkers are only effective for a brief window of time (Liu et al., 2022). For instance, with conditions like arrhythmia, various forms of rapid supraventricular arrhythmias occur infrequently, only happening once every few hours or less, with these episodes being of short duration, often just seconds or minutes. In the case of epilepsy patients, seizures represent merely 0.01% of their overall life span. The proposed event-driven chip employs an extreme compressed binary neural network (BNN) from the baseline model for continuous event detection and a moderate convolutional network for precise biomarker detection. The binary neural network can be achieved through quantize weights to binary values as mentioned in Section 2. As it only requires 1-bit weights and simple logic operations, the reduction in computational demands significantly decreases the required power consumption. The BNN is engineered to preserve a high level of sensitivity to guarantee that no potential onset goes undetected. However, due to the inherent limitations in the classification capabilities of the BNN, the rate of false alarms cannot be assured with BNN alone. To reject false alarms, a high-precision convolutional neural network will be activated after an event has been detected by the BNN. The convolutional neural network helps eliminate additional false alarms, ensuring overall low false alarm rate at system level. As illustrated in Figure 4, when employing the BNN model for event detection, the system successfully filters out most false alarms and operates in a low-power state. The CNN mode is briefly engaged to confirm alarms that surpass the event threshold. The BNN model predominantly governs the system’s power consumption, whereas the CNN model dictates the rate of false alarms. To seamlessly integrate the BNN and CNN networks and conserve silicon area, the chip has been designed in a reconfigurable fashion without using any multiplier.

### 3.1 Neural signal processing architecture

**Figure 5:**
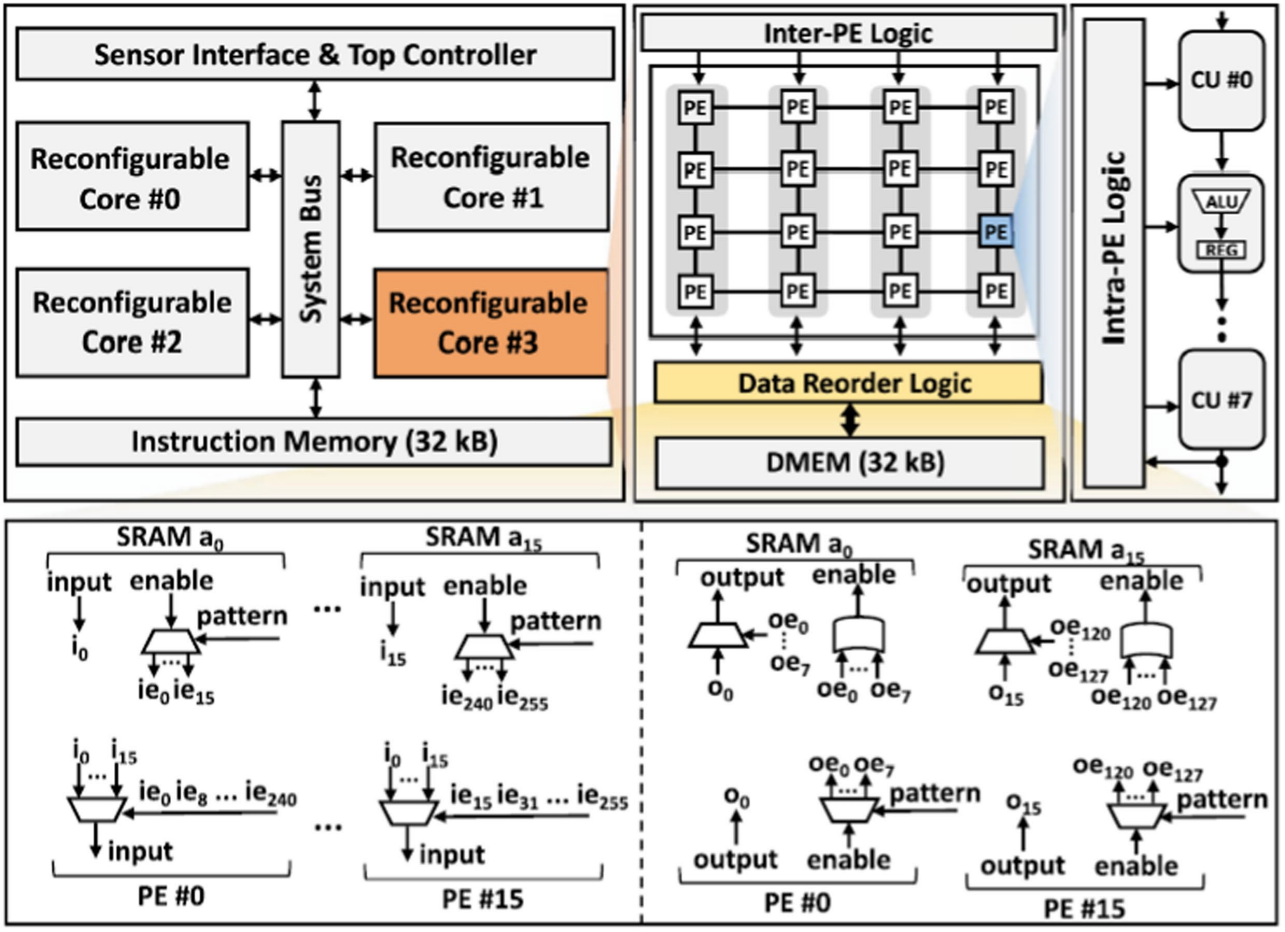
The architecture of the proposed event-driven neural signal processor and the structure of data reorder logic.

Figure 5 provides the architecture of the proposed processor. It consists of four reconfigurable cores interconnected via a system bus. The sensor interface is responsible for receiving external input neural signals. The top controller fetches instructions from a 32 kB instruction memory through the system bus and controls the overall system while streaming data from the sensor interface to the four reconfigurable cores. Each core includes an array of Processing Elements (PEs), data reorder logic, 32 kB local data memory, and inter-PE logic that governs the behavior of the PE array. The data reordering mechanism contains two parts, the input data reorder and the output data reorder as illustrated in Figure 4. The input order logic connects 16-source ports from the data SRAM within the core to 16-destination ports at the PEs. Data is received at each source port from a designated SRAM address (e.g., i_0_), with an enable signal (e.g., ie_0_) activated based on the decoded instruction pattern. This signal guides the routing of input data to any of the PEs for subsequent processing. For the output reorder logic, 16-source ports from the PEs are linked to 16-destination ports at the data SRAM. These ports handle the processed data output from the PEs (e.g., o_0_), with each piece of output data linked to an output enable signal (e.g., oe_0_). This signal specifies which data bits should be stored back into the SRAM at the appropriate addresses. Each PE consists of eight pipelined Computation Units (CU) and intra-PE logic controlling the pipeline configuration, control signals, and inputs for each CU. The proposed processor is designed to support various sizes of CNN and BNN computation. The limitation on model size is determined by the memory requirement for the maximum intermediate layer if maps values and the weights.

**Figure 6:**
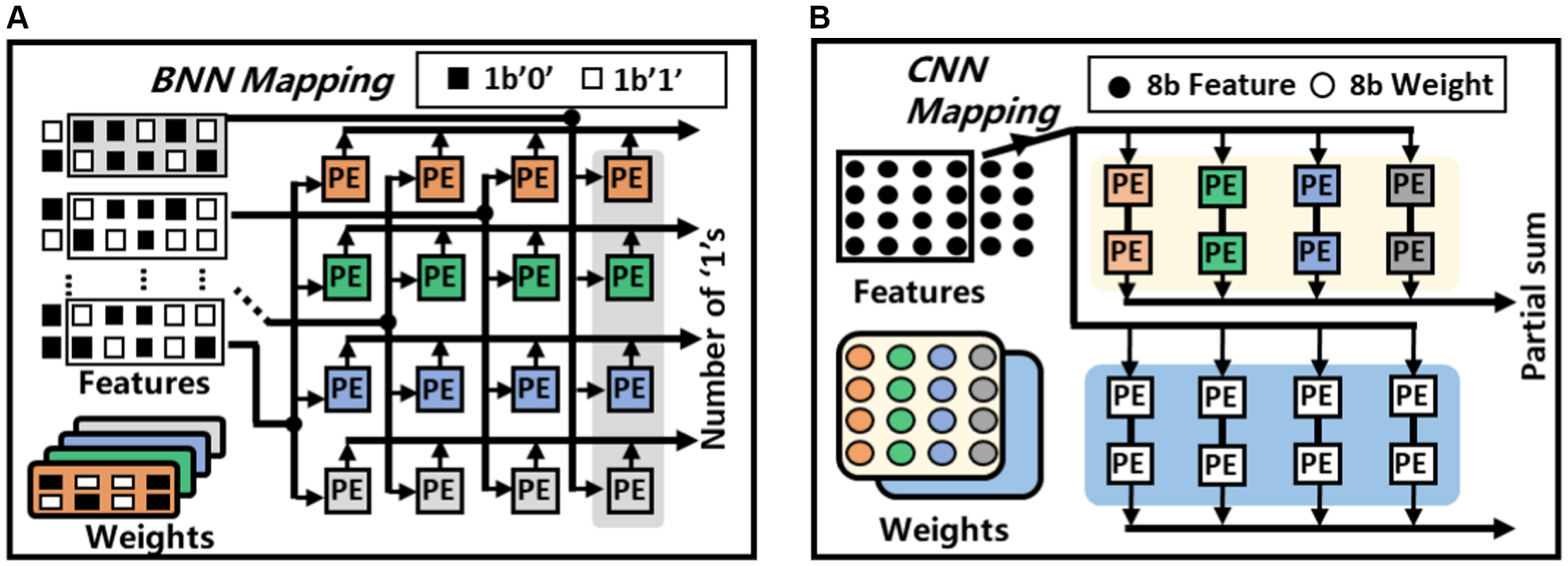
Weight and feature mapping methods of the proposed architecture, **(A)** BNN mapping, **(B)** CNN mapping.

Figure 6 shows the BNN and CNN mapping of the proposed architecture. During always-on event detection mode, each PE can work independently to compute the multiple-accumulation result of eight pairs of binary numbers. Taking a 4-channel 2 × 4 convolution operation as an example, every 2 × 4 binary weight kernel can be treated as an 8-bits integer. Every such 8-bit integer are copied to a row of PEs. The feature maps are grouped in the same fashion and are broadcast in a column-wise fashion to the PEs. Then every PE performs XOR and Popcount operations to generate the partial sum of different kernels. After broadcasting the entire feature map, the first set of BNN convolution operations are completed, and the feature map can be replaced for the next round of operations. Upon detecting an event with the BNN configuration, the chip can be reconfigured to CNN mode for precise classification which involves more complex multiplication operations. As shown in Figure 6B, two PEs are interconnected, every four 8-bits values in the weight kernel are placed in the two PEs of the same group, thus, 16 values in one weight kernel can be placed in the first two rows of the PEs. The 16 values in the other weight kernel can be mapped into the eight PEs in the next two rows. The CNN feature map will be grouped every four values in the order of computation. Subsequently, they are placed together into the four PEs with placed weights. Simultaneously, these feature maps are also broadcast to the four PEs where another weight kernel is placed. These feature maps are passed forward in a chain until the partial sums of the 1 × 4 vectors inner product is calculated. The partial sums computed by multiple PEs will be passed to other PE arrays for accumulation to obtain the final convolutional operation results.

### 3.2 Reconfigurable processing element design

**Figure 7:**
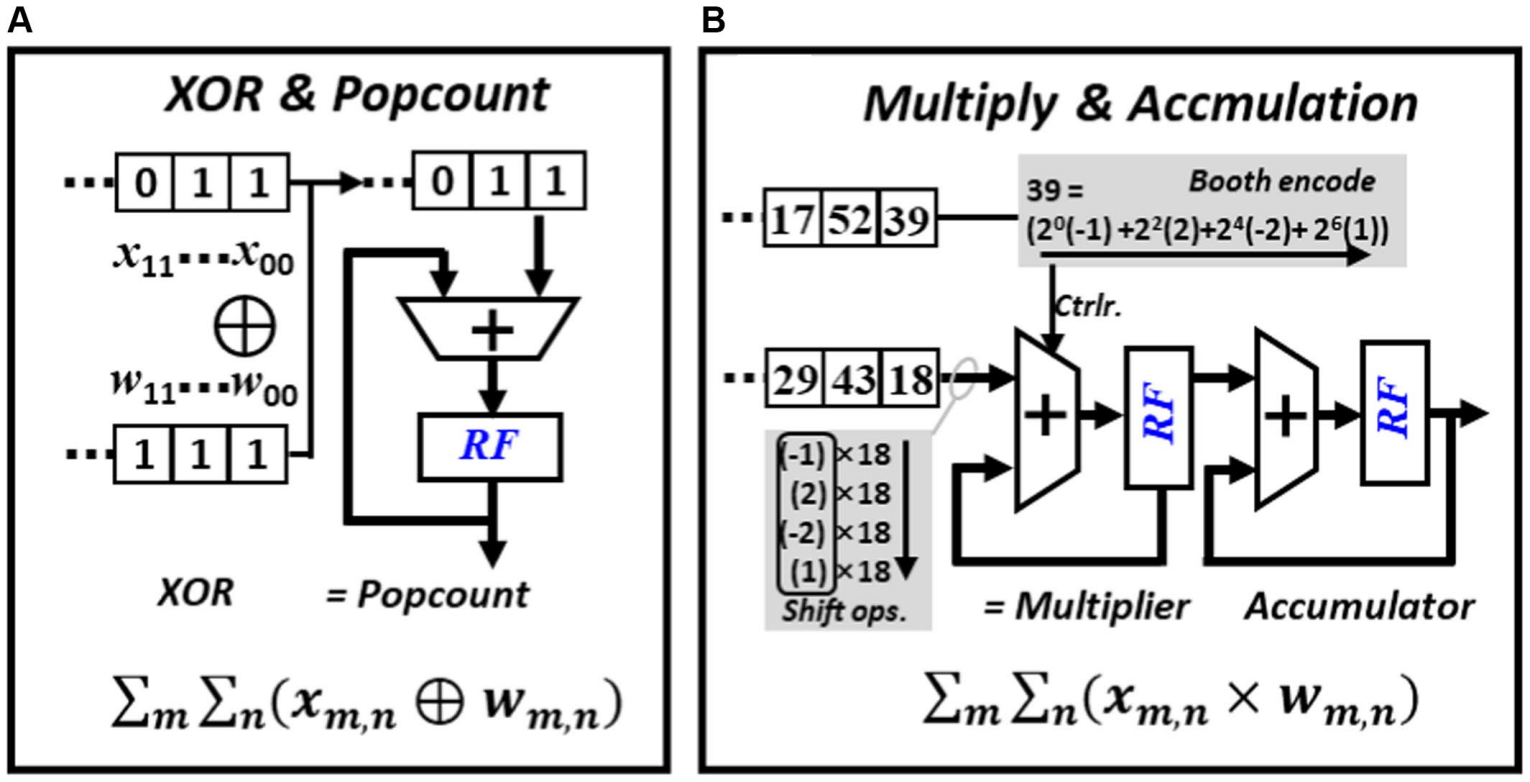
Major operations required in BNN and CNN inference, **(A)** PE configured to perform popcount operation, **(B)** PE configured to perform booth encoded multiplication.

The computation of BNN mainly involves XNOR-popcount operations. As shown in Figure 7A, during inference, multiple bits from weights and activations are grouped into two sets. The XNOR operation is performed based on these two groups of values, and then the number of resulting “one” are counted. The popcount operation can be completed by right shift the result of XNOR in each cycle and add the extracted least significant bit (LSB) to the left spare position of the register. The computation of CNN mainly involves multiplication and accumulation operations. To mitigate the usage of area and power consuming multipliers, all multiplication operations in this work are replaced with booth encoded accumulations. As shown in Figure 7B, an 8-bit multiplier can be encoded into four values and each represent a value in [−1, −2, 0, 1, 2]. The final multiplication operation can be implemented by shifting or adding the multiplicand. Unlike conventional multiplication where the multiplier and the multiplicand can vary frequently, in CNN inference, either the feature map or the weight can stay stationary during the multiplication process.

**Figure 8:**
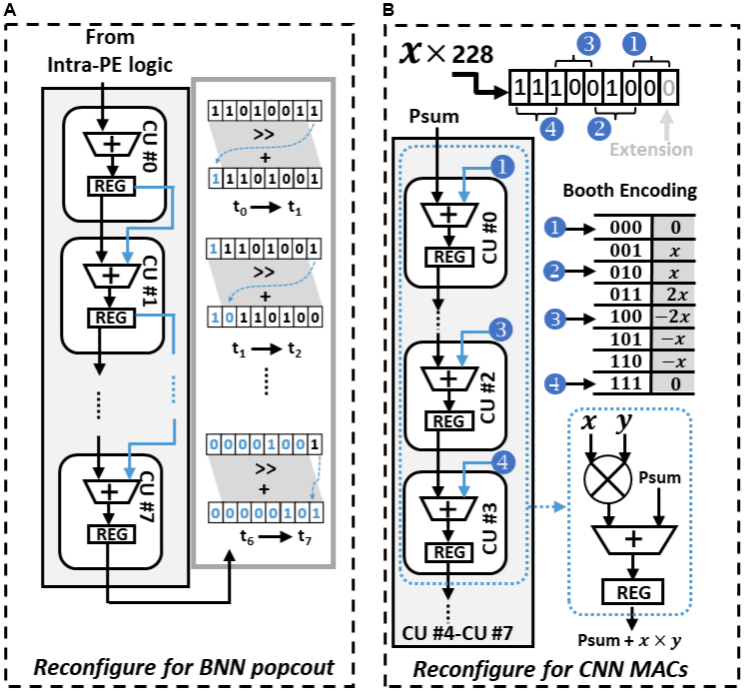
Reconfigure PE to perform different operation, **(A)** PE configured to perform popcount operation, **(B)** PE configured to perform booth encoded multiplication.

Figure 8A shows the BNN circuit-level paradigm of the PE, it is configured to facilitate the popcount operations. In this mode, binary weights and feature maps originally represented as −1 or 1 are converted to 0 or 1. The conversion process begins with the PE employing its internal logic to compute the XNOR result from the weights and input feature maps. The CUs within each PE are interconnected in a sequential manner to execute popcount operations. Each XNOR result undergoes a one-bit shift to the right, and the bit that is shifted out is added to the rightmost spare bits in the register. These spare bits serve as a temporary storage to tally the number of “1”s counted. In the following cycle, the subsequent CU carries on with this operation, continuously updating this temporary count until all bits have been accounted for. This repetitive process enables the efficient computation of popcounts during BNN inference. As illustrated in Figure 8A, after this interactive process, the register’s value updates to “00000101,” indicating that there are five “1”s in the original XOR result of “11010011.”

In the CNN mode, the PE undergoes a reconfiguration to operate as a Multiply-Accumulate (MAC) unit using Booth encoding, eliminating the need for a multiplier. Figure 8B demonstrates how this mode facilitates the MAC operation at the circuit level, where two 8-bit input numbers, a multiplicand “x” and a multiplier “y,” interact to complete the MAC operation. The process begins with the extension of the least significant bit (LSB) of “y,” transforming it into a 9-bit multiplier. This extended multiplier “y” is then divided into four 3-bit Booth multipliers. Each of these Booth multipliers encodes the multiplicand “x” using Booth encoding, resulting in four Booth products. These four Booth products are subsequently assigned to four consecutive Computational Units (CUs) in a specific order. In contrast to the BNN mode, where one of the inputs of each CU’s ALU is connected to the Least Significant Bit (LSB) of the preceding CU’s register, one ALU input is now linked to the predetermined Booth products values. Within each CU, the Booth products are added with the other input of the ALU, namely the old partial sums (psums) obtained from the previous CU. This addition yields new partial sums, which are then stored in the CU’s register and subsequently transmitted to the next CU. This iterative method of incorporating Booth products with propagated partial sums (psums) throughout the chain of Computational Units (CUs) enhances the efficient execution of Multiply-Accumulate (MAC) operations. In contrast to general multiplication scenarios where both the multiplicand and multiplier are arbitrary, in convolutional operations, the multiplicand remains constant as it represents the kernel value. Consequently, the Booth products can remain unchanged throughout the computation of an entire kernel.

**Figure 9:**
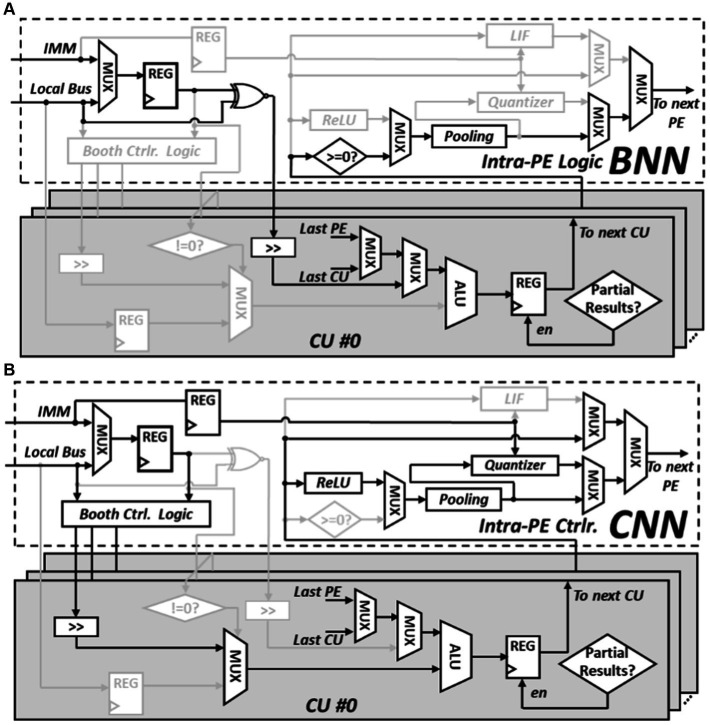
Reconfigurable details of PE, **(A)** intra-PE connection in BNN configuration **(B)** intra-PE connection in CNN configuration.

The intra-PE logic and the CU circuit diagram is shown in Figure 9. In BNN mode, the top controller oversees the operations and help read binary weight from data memory and reorganize weights into suitable weight groups through data reorder logic within the reconfigurable core. Once organized, these weights are written into the registers using the intra-PE logic and waiting for the input feature map to perform XNOR operations. The results of these XNOR operations are then sent to the first CU to start the popcount calculations. The final CU within the PE is responsible for propagating the results back to the intra-PE logic. If the computation involves a partial convolution operation, the outcome is forwarded to the next connected PE. However, if it’s a complete convolution operation, the final result is achieved by applying the sign activation function and the max pooling module within the intra-PE logic.

During the CNN mode, multipliers are fetched from the data memory and stored in the registers of the intra-PE logic. These multipliers serve the purpose of encoding the multiplicand using Booth control logic. The encoded Booth products are then directed to the respective CU. Depending on if the output is partial sum or final results, the finial CU will direct the data to the next PE for further accumulation or necessitates the execution of the Rectified Linear Unit (ReLU) activation function and the max pooling operation by the respective modules. The data sent to the data memory needs to be quantized to 8-bits. The quantization process is managed by the quantizer module, and its configuration is determined by the immediate instructions decoded by the top controller.

## 4 Experiments and evaluation results

Evaluations of this study are conducted in terms of model classification accuracy, false alarm rate and chip power consumption. Experimental setup including graphic user interface (GUI), PCB and FPGA test board are designed to facilitate the testing of the proposed algorithm and the chip.

### 4.1 Closed-loop control performance evaluation

This paper employs three datasets to assess the performance of the proposed model: the American Epilepsy Society Seizure Prediction Challenge (AES) intracranial electroencephalogram (iEEG) dataset (Brinkmann et al., 2016), the Melbourne University iEEG dataset (Kuhlmann et al., 2018a), and the CHB-MIT electroencephalogram (EEG) dataset (Goldberger et al., 2000). The AES dataset comprises iEEG recordings collected from five dogs and two human subjects. The dog data was sampled at a rate of 400 Hz, with 16 electrodes used for four dogs and 15 electrodes for one dog (Dog5). The human subjects’ iEEG data were sampled at 5,000 Hz, using 15 electrodes for one patient and 24 electrodes for the other patients. The Melbourne University dataset, accessible through the Melbourne-University AES-MathWorks-NIH Seizure Prediction Challenge, contains iEEG signals from three patients. Each patient had 16 electrodes implanted, and all measurements were sampled at a frequency of 400 Hz. The CHB-MIT dataset comprises EEG data from 22 patients, recorded over multiple days, resulting in a total of 637 recordings, including 163 seizures. Most measurements were obtained using 23 fixed electrodes, and the sampling rate for all subjects was 256 Hz. There were variations in the data, including the number of seizure events and data length. For seizure prediction task, it is essential to consider the Seizure Prediction Horizon (SPH) and the Preictal Interval Length (PIL) during the data preprocessing stage (Yang and Sawan, 2020; Wang et al., 2021). The SPH denotes the time interval between the onset of a seizure and the preictal phase, while the PIL quantifies the duration of the preictal state. In the case of the two iEEG datasets, we configured the SPH and PIL to be 5 min and 1 h, respectively. Conversely, for the CHB-MIT EEG dataset, these values were set to 5 and 30 min, respectively. In accordance with the specified SPH and PIL parameters, the training data is segregated into preictal and interictal samples. To address the data imbalance between these two sample types, preictal samples are extracted with 5-s overlaps, while interictal samples are extracted without any overlaps.

**Figure 10:**
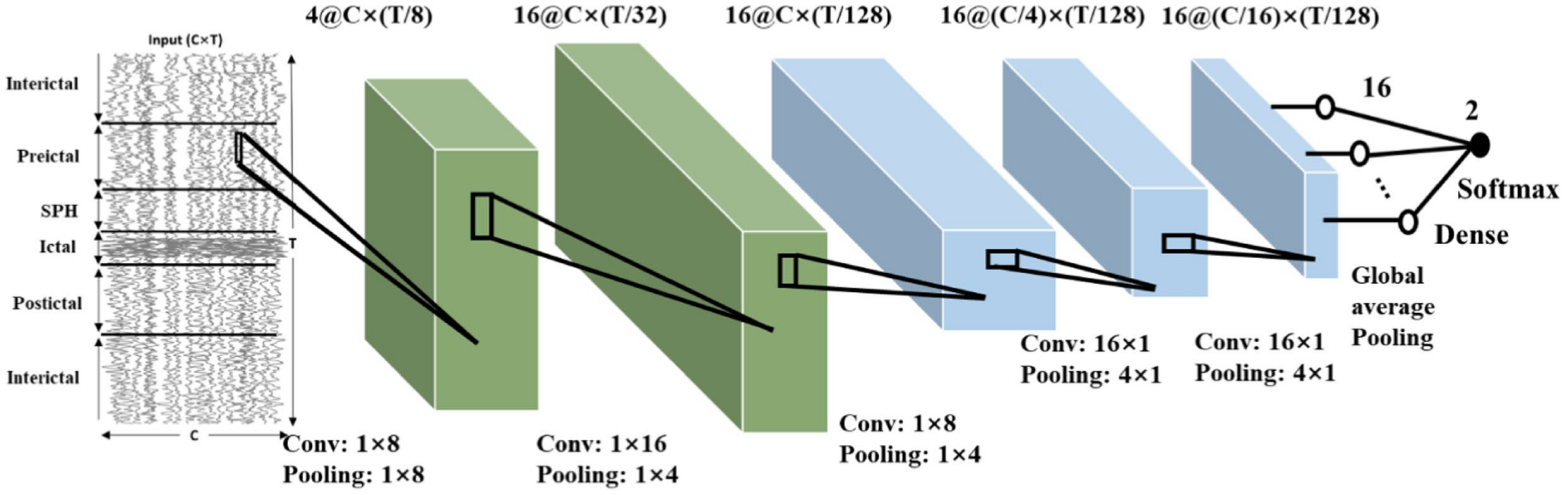
The obtained network structure based on the constraints and CHB-MIT dataset.

Following the use of the proposed method in Section 2, we can obtain a baseline model and quantize the model to 8-bit quantization or binary models. The CNN model obtained with CHB-MIT dataset is shown in Figure 10. The architecture comprises five convolutional blocks, each consisting of a convolutional layer followed by a pooling layer. A ReLU activation function is applied after the convolution. The output is generated using a global average pooling layer, followed by a dense layer applying the Softmax function. The dimensions of the convolution and pooling for each layer are also depicted in Figure 10.

Due to the difference between sampling rate and number of channels, the network architecture can vary based on different dataset. Throughout the training process, we employ the leave-one-out cross-validation method to mitigate the risk of potential overfitting and helps ensure the robustness of our model’s performance. Critical metrics, including sensitivity, false alarm rate, model size is presented in Table 1. Our 8-bit quantization model achieved overall better performance when compared with previous reported top-performing models in different datasets. The 8-bit model can outperform other models in both sensitivity and false alarm rate. Although the binary model may not achieve a low false alarm rate comparable to other models, it significantly reduces computational and memory demands.

**Table 1:** Comparison with closed-loop control algorithms for seizure prediction.

Method	Database	Sensitivity (%)	False alarm rate(h^−1^)	Model size
Truong et al. (2018)	CHB-MIT	81.2	0.16	0.8 MB
	AES	75.0	0.21	0.8 MB
Eberlein et al. (2018)	CHB-MIT	96.3	0.02	1 MB
	AES	91.9	0.10	1 MB
	Melbourne	74.0	0.21	1 MB
Lawhern et al. (2018) (EEGNet-8,2)	CHB-MIT	92.9	0.07	17.8 kB
	AES	84.5	0.12	22.3 kB
	Melbourne	77.7	0.30	22.3 kB
This work (8-bit, CNN)	CHB-MIT	99.6	0.01	23.5 kB
	AES	91.8	0.09	20.2 kB
	Melbourne	85.2	0.12	15.2 kB
This work (1-bit, BNN)	CHB-MIT	99.2	0.81	13.8 kB
	AES	98.7	0.94	9.5 kB
	Melbourne	95.3	1.4	6.5 kB

### 4.2 Chip implementation and performance

**Figure 11:**
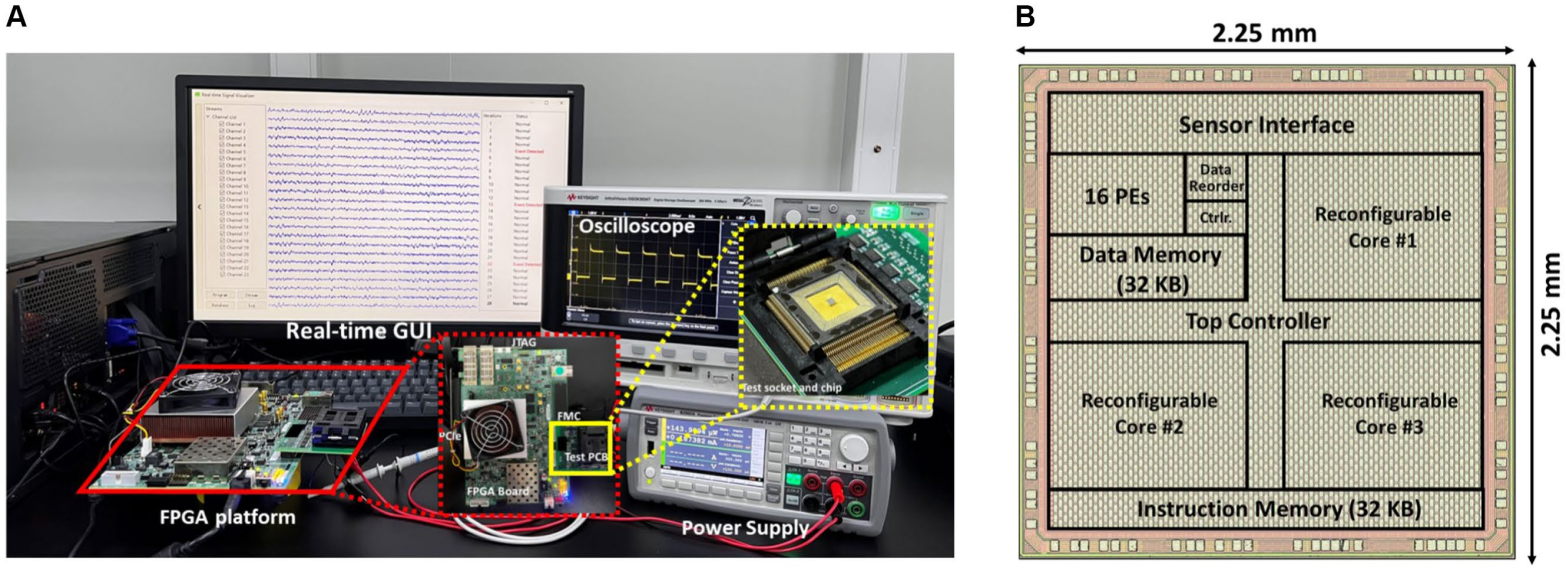
Test system and chip photograph, **(A)** test system with GUI, FPGA platform and PCB board, **(B)** microphotograph of the proposed chip.

**Figure 12:**
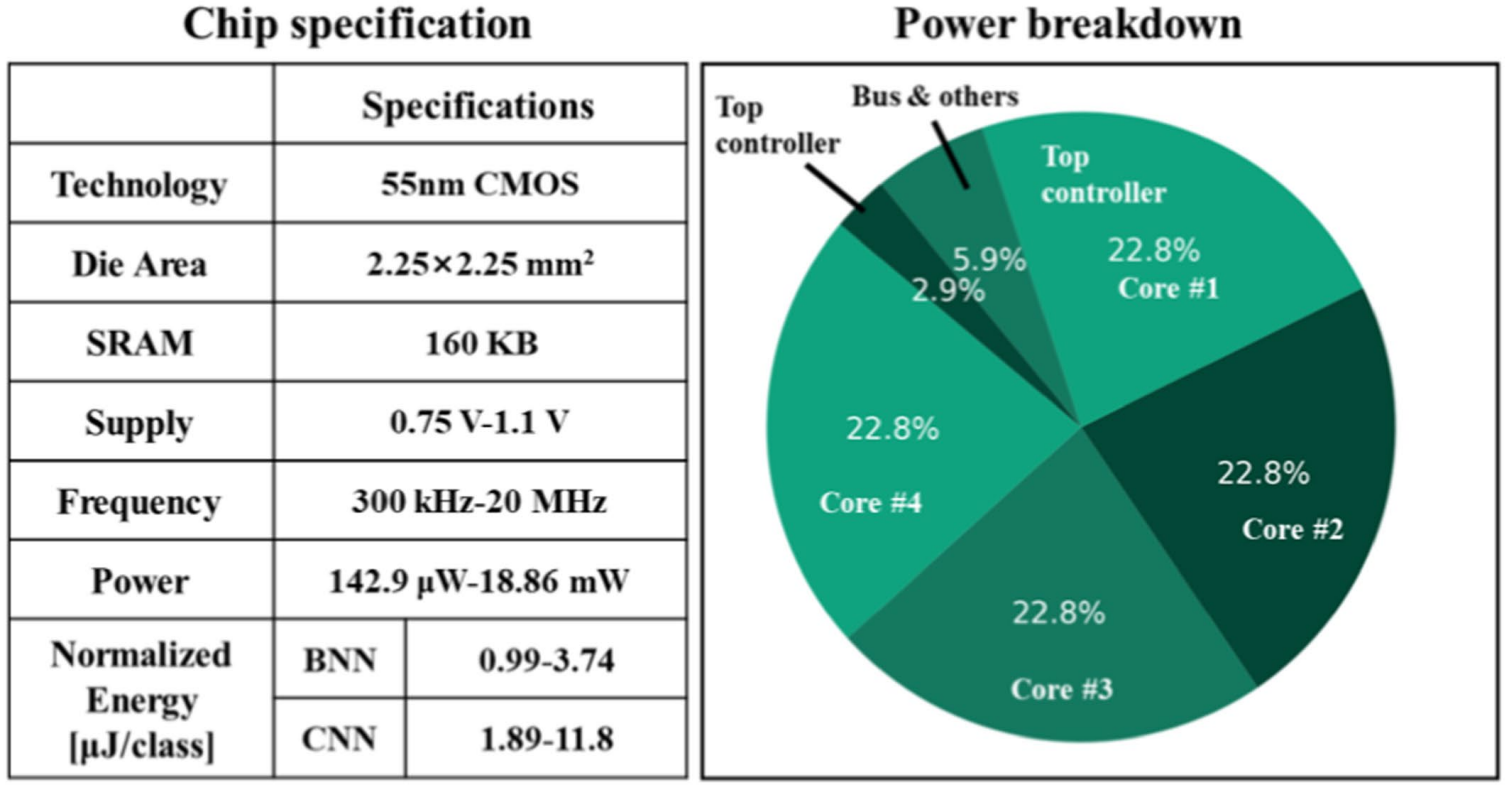
Performance summary and power breakdown of the proposed processor.

Figure 11A illustrates the prototype system designed for evaluating the proposed processor. The test system consists of several essential components, including an oscilloscope, power supply, a testing printed circuit board (PCB) that links the FPGA to the chip, and a real-time graphical user interface (GUI) on a desktop computer (PC). The PCB is interconnected with the FPGA via the FMC interface, and the FPGA communicates with the host PC through the PCIe interface. The GUI acts as the control central of the demonstration system, enabling various functions such as displaying neurological signals, loading instructions and data onto the chip, and collecting and presenting real-time calculation results generated by the chip. During operation, the GUI retrieves and streams electrophysiological signals from the database to the FPGA board via the PCIe interface. The FPGA board then executes the PCIe protocol, decoding the incoming data to align with the standard sensor interface format. Final, the FPGA retrieves the processed data from the chip and then these results are displayed on the GUI. Figure 11B displays the chip photograph along with its performance summary. The chip was fabricated using SMIC 55 nm CMOS technology and occupies an area of 2.5 × 2.51 mm^2^. It can operate within a clock frequency range from 300 kHz to 20 MHz and with a supply voltage range of 0.75 to 1.1 volts. During the seizure prediction task, power consumption measures at 142.9 μW with a 300 kHz frequency and 0.75 V supply voltage, while it reaches 18.86 mW at a 20 MHz frequency and 1.1 V supply voltage. Operating at a frequency of 20 MHz, the energy consumption per inference is 3.74 μJ for BNN and 11.8 μJ for CNN. When the frequency is reduced to 300 kHz, the energy consumption decreases to 0.99 μJ for BNN operations and 1.89 μJ for CNN operations. A breakdown of the power distribution for the chip is presented in Figure 12. The four reconfigurable cores accounting for 91.2% of total power consumption. The top RISC, system bus and other components contribute to 2.9, 5.9% of the power consumption, respectively. The chip achieves peak energy efficiency at 300 kHz and 0.75 V. Table 2 provides comparison between the proposed chip and existing state-of-the-art works. This chip incorporates event-driven processing, enabling it to effectively handle biomarker detection with low-power consumption. The proposed chip represents the first implementation of event-driven processing through a reconfigurable design featuring two neural networks for closed-loop neuromodulation control.

**Table 2:** Comparison with closed-loop control algorithms for seizure prediction.

Method	This work	JSSC’23 (Hsieh et al., 2022)	JSSC’2019 (Huang et al., 2019)	JSSC’2018 (O’Leary et al., 2018)
Technology	55	40	40	130
Area	5.06	1.96	4.5	3.3
On-chip Memory	160 kB	5 kB	NA	NA
Classifier	BNN + CNN	SVM	SVM	SVM
Frequency	300 kHz–20 MHz	6 MHz	130 K, 65 K	10 M
Application	Seizure prediction	Seizure prediction	Seizure detection	Seizure detection
Inference consumption	0.99–11.8 μJ	96.2 nJ	170.9 μJ	168.6 μJ
Power consumption	142.9 μW–18.86 mW	2.3 mW	1.9 mW	0.67 mW

## 5 Conclusion

In this paper, we propose an algorithm and integrate circuits co-design strategy to close the loop of implantable neuromodulation devices. Low memory and computation demand convolutional neural network is first searched and compressed with architecture search and quantization techniques. The obtained network architecture achieves low false alarm rate of 0.1/h and high sensitivity while reducing model size about 50% when compared to state-of-the-art models. A dedicated neural signal processor that can implement the networks is designed and fabricated in 55 nm technology. With the event-based processing scheme, the chip can switch between low-power consumption event detection mode and high precision classification mode to maintain both real-time performance and low-power consumption. The chip can operate under 0.75 V supply voltage and 300 kHz clock frequency with only 143 μW power. In summary, the proposed algorithm and integrated codesign strategy can offer versatility, high accuracy, and outstanding energy efficiency to close the loop for neuromodulation applications. While our proposed algorithm and integrated co-design strategy showcase significant improvement toward energy efficiency and accuracy, we acknowledge challenges such as catastrophic forgetting and the need for meta-learning capabilities that warrant further investigation in the field of closed-loop neuromodulation. Additionally, clinical validation is an essential next step in our continued research efforts.

## Data availability statement

The original contributions presented in the study are included in the article/supplementary material, further inquiries can be directed to the corresponding author.

## Author contributions

JY: Writing – original draft, Writing – review & editing, Conceptualization. SZ: Writing – review & editing, Data curation, Software. JW: Software, Writing – review & editing. SL: Writing – review & editing, Validation, Software. QH: Writing – review & editing, Validation. MS: Funding acquisition, Supervision, Writing – review & editing, Project administration.
